# The expression of metastasis-associated in colon cancer-1 and KAI1 in gastric adenocarcinoma and their clinical significance

**DOI:** 10.1186/s12957-016-1033-z

**Published:** 2016-10-28

**Authors:** Guoyu Lu, Lei Zhou, Xiaohua Zhang, Bo Zhu, Shiwu Wu, Wenqing Song, Xiaomeng Gong, Danna Wang, Yanyan Tao

**Affiliations:** 1 Department of Emergence, The First Affiliated Hospital of Bengbu Medical College, No.287, Changhuai Road, Bengbu, China; 2 Department of Pathology, the First Affiliated Hospital of Bengbu Medical College, No.287, Changhuai Road, Bengbu, China; 3 Department of Pathology, Bengbu Medical College, No.2600, Donghai Street, Anhui Province, China

**Keywords:** Gastric adenocarcinoma, MACC1, KAI1, Metastasis, Prognosis

## Abstract

**Background:**

The most common reason for malignant tumor treatment failure is recurrence and metastasis. Metastasis-associated in colon cancer-1 (MACC1) was originally identified as a metastatic and prognostic biomarker for colon cancer and later other solid tumors. Kangai 1 (KAI1), a marker of suppressor of metastasis, is also associated with metastasis and poor prognosis in many tumors. However, the prognostic value of either MACC1 or KAI1 in gastric adenocarcinoma (GAC) is unclear. In this study, we explored the relationship between MACC1 and KAI1 expression, as well as their respective correlation with clinicopathological features, to determine if either could be helpful for improvement of survival prognosis in GAC patients.

**Methods:**

The expression levels of both MACC1 and KAI1 in 325 whole-tissue sections of GAC were examined by immunohistochemistry. Clinical data was also collected.

**Results:**

MACC1 was significantly overexpressed in GAC tissues when compared to levels in normal gastric tissues; KAI1 was significantly down-expressed in GAC tissues when compared to levels in normal gastric tissues. Investigation of association between MACC1 and KAI1 protein levels with clinicopathological parameters of GAC indicated association between the expression of each with tumor grade, lymph node metastasis, invasive depth, and TNM stages. The overall survival time of patients with MACC1- or KAI1-positive GAC tumors was significantly shorter or longer than that of those who were negative. Importantly, multivariate analysis suggested that positive expression of either MACC1 or KAI1, as well as TNM stage, could be independent prognostic factors for overall survival in patients with GAC.

**Conclusions:**

MACC1 and KAI1 may represent promising metastatic and prognostic biomarkers, as well as potential therapeutic targets, for GAC.

## Background

There were approximately 950,000 new stomach cancer cases and 720,000 deaths that occurred in 2012 worldwide [1]. In general, China is one of the highest incidence countries worldwide [1]. Gastric adenocarcinoma (GAC) accounts for approximately 90 % of all diagnosed stomach cancers. It was also one of the most frequent causes of cancer-related deaths. The 5-year survival rate is less than 5 % for GAC patients with distant metastasis, as well as is less than 60 % for patients with only localized malignancies [2]. In China, the majority of patients diagnosed with GAC have advanced stages of disease and are unsuitable for curative surgery.

Tumor recurrence and metastasis are the most common cause of treatment failure. Tumor recurrence and metastasis involve in multiple steps with a high degree of complexity and require the contribution of many molecules. Metastasis-associated in colon cancer-1 (MACC1) is a gene which contributes to these processes. MACC1 was first identified in colon cancer in 2009 and was bound to the promoter of the mesenchymal-epithelial transition (MET) gene to control its transcriptional activity [3, 4]. In vitro, MACC1 may drive proliferation, migration, invasion, and dissemination [5]; in vivo, it may regulate gene transcriptionally for metastasis, such as tyrosine kinase MET [5–7]. Furthermore, accumulating evidence has indicated that MACC1 should contribute to apoptosis and epithelial-mesenchymal transition (EMT) via hepatocyte growth factor/mesenchymal-epithelial transition (HGF/MET) pathways [8]. MACC1 was also considered as a decisive driver for metastasis and tumorigenesis [9]. MACC1 was also an independent prognostic factor for colon cancer [3, 5]. Now, more and more studies have demonstrated that MACC1 could also be a metastatic and prognostic factor for various human cancers, including pancreatic [10], liver [11], lung [12], ovary [13], breast [14], gastric [8], malignant glioma [15], and cervical carcinoma [16].

Kangai 1 (KAI1) was first identified as a suppressor of metastasis gene in prostate carcinoma [17]. KAI1 protein which is located in human chromosome 11p11.2 is a member of the transmembrane 4 superfamily (TM4SF). KAI1 can regulate signal transduction both cells to cells and cells to extracellular matrix (ECM) [18] and involve in some fundamental biological processes such as fusion, migration, adhesion, fertilization, differentiation, and invasion [19, 20]. Accumulating evidence has demonstrated that decreased or lost KAI1 expression should associate with metastasis and prognosis in various tumors, including laryngeal carcinoma [20], prostate carcinoma [19, 21], breast carcinoma [22], lung carcinoma [23], gastric carcinoma [24], colon carcinoma [25], and hepatocellular carcinoma [26].

The involvement of MACC1 and KAI1 in the recurrence and metastasis of GAC suggests that they should be valuable biomarkers for measuring cancer progression and developing higher accurately therapeutic targets. To our knowledge, a correlation between MACC1 and KAI1 in GAC has not yet been reported. In this study, we detected the association between MACC1 and KAI1 expression in patient cancer tissues as well as compared their expression with clinicopathology, metastasis, and prognosis of GAC.

## Methods

### Biopsy specimens

GAC tissues and adjacent noncancerous gastric tissues were collected at the Department of Pathology of the First Affiliated Hospital of Bengbu Medical College, from January 2008 to December 2010. Patients who had received preoperative chemo- or radio-therapy were excluded. All tissue specimens were obtained with patient consent, and the research was approved by the ethical committee of Bengbu Medical College and conducted in accordance with the ethical guidelines of the Declaration of Helsinki. The adjacent noncancerous gastric tissues were removed from the same patient, avoiding necrotic tissue, and from surrounding gastric tissue at least 5 cm away from the carcinoma edge. The research group consisted of 325 patients, 214 males and 111 females, aged from 26 to 78 years; the average age was 57.7 ± 10.9 years. All patients who had completely clinical, pathological, and follow-up (at 8-month intervals by phone, mail, or email) data were sporadic cases. Overall survival (OS) time was collected from surgery to death or December 2015 (mean OS time 42.0 months; range 8–95 months). Tumor node metastasis stage was evaluated according to the 7th edition of the American Joint Committee on Cancer (AJCC). Grade of tumor differentiation was according to the World Health Organization (WHO) standard.

Please contact author for data requests.

### Immunohistochemistry

All GAC and corresponding normal gastric tissues were fixed in 10 % buffered formalin and embedded in paraffin. Then continuous 4-μm-thick tissue sections were cut. Subsequently, all sections were deparaffinized and dehydrated with xylene, graded ethanol, and washed for 10 min in PBS (pH 7.2). Immunohistochemistry was performed according to the Elivision Plus detection kit instructions (Lab Vision, USA). Endogenous peroxidase activity was blocked by incubation of sections in methanol containing 3 % H_2_O_2_ for 10 min at room temperature, then placed in citrate buffer (pH 6.0) and heated to 95 °C for 30 min for antigen repair. After several washes in PBS, the sections were quenched with goat serum for 20 min at room temperature, then incubated with rabbit polyclonal antibody against human MACC1 (Santa Cruz Biotechnology, Santa Cruz, CA, USA) or mouse monoclonal antibody against human KAI1 (Abcam, Cambridge, MA, USA) for 1 h at 37 °C. All sections were counterstained with hematoxylin, dehydrated, air-dried, and mounted. Negative controls were prepared by deleting primary antibodies from the staining procedure. MACC1-positive staining was mainly confined in the cytoplasm of cancer cells, and KAI1-positive staining was mainly confined in the membrane and cytoplasm of cancer cells.

### Evaluation of staining

Staining results were evaluated by two experienced pathologists who were blind to the clinical data and assessed by semi-quantitative scores. Because of intratumoral heterogeneity of antibody expression, we randomly chose ten visual fields from different areas of each section of GAC. If there was a disagreement, the pathologists would reexamine the immunostaining and reach a consensus [27–29]. To assess MACC1 and KAI1 expression, both the extent and intensity of immunostaining were thought [27]. The staining extent score was graded as follows: none, 0; weak, 1; moderate, 2; and strong, 3. The intensity of positive staining was graded as follows: <10 %, 1; 11–50 %, 2; 51–75 %, 3; and >75 %, 4. Then the score was determined by multiplying the extent and intensity of immunostaining to reach a range of scores from 0 to 12. For tumors that were positive for both MACC1 and KAI1, an average of the final of each sample was taken. Immunostaining was thought positive when the score was ≥3.

### Statistical analysis

Relationship between either MACC1- or KAI1 protein expression and clinicopathological parameters were compared using Fisher’s exact test or chi-square test. The correlation between MACC1 and KAI1 expression was compared using Spearman’s coefficient test. The effects of MACC1 and KAI1 expression on OS time were determined using Kaplan-Meier method for univariate analysis. Independent prognostic indicators were determined using the Cox regression model for multivariate analysis. The association between the positive expression of either MACC1 or KAI1 and clinicopathological parameters was determined using SPSS 19.0 software for Windows (Chicago, IL). A value of *P* < 0.05 was determined as statistically significant.

## Results

All GAC patient clinicopathological characteristics could be seen in Table 1.

**Table 1:** Patients characteristics

Patients characteristics	Frequency (*n*)	Percentage (%)
Gender		
Male	214	65.8
Female	111	34.2
Ages		
<58	140	43.1
≥58	185	56.9
Gross type		
Polypoid	37	11.4
Ulcerative	220	67.7
Invasive	68	20.9
Location		
Antrum	163	50.2
Cardia	118	36.3
Pylorus	44	13.5
Size		
*D* < 4.0 cm	75	23.1
4.0 cm ≤ *D* < 8.0 cm	211	64.9
8.0 cm ≤ *D*	39	12.0
Depth of invasion		
Submucosa	21	6.5
Subserosa	100	30.8
Visceral peritoneum	184	56.6
Adjacent structures	20	6.2
Tumor grade		
Well	47	14.5
Moderate	204	62.8
Poor	74	22.8
Lymph node metastasis		
No	178	54.8
Yes	147	45.2
TNM stage		
I and II	153	47.1
III and IV	172	52.9

### Expression of MACC1 and KAI1 in GAC and their association with clinicopathology

**Fig. 1:**
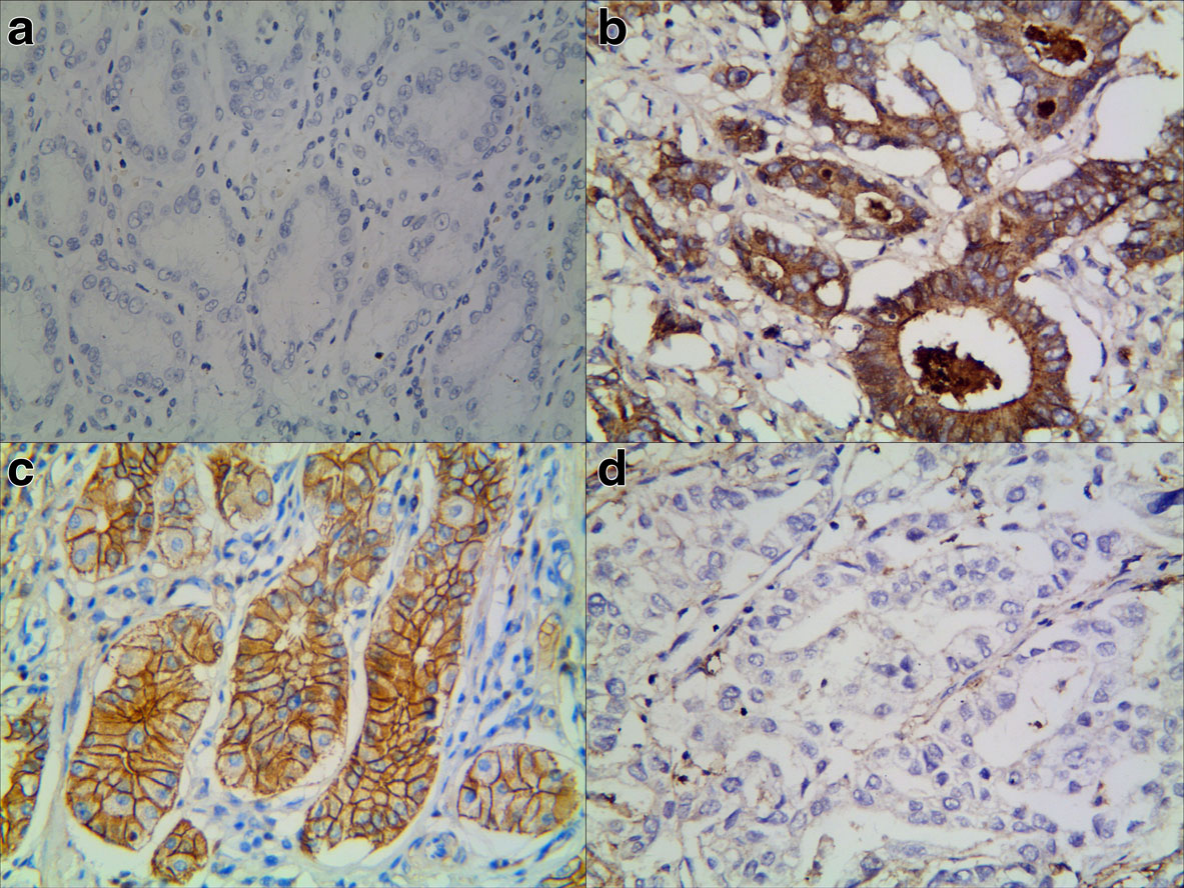
Representative results of MACC1 and KAI1 in gastric adenocarcinoma and control group. **a** Control gastric epithelial cells not expressing MACC1. **b** MACC1 predominantly localized in the cytoplasm in moderate grade of gastric carcinoma (MACC1 ×400). **c** Control gastric epithelial cells expressing KAI1 in the membrane and cytoplasm. **d** Moderate grade of gastric carcinoma cells not expressing KAI1 (KAI1 ×400) (**b** and **d** are the same GAC patient’s slice)

To assess the contributions of MACC1 and KAI1 to GAC, their expression levels were evaluated in both GAC and normal gastric tissue slides using immunohistochemistry. MACC1-positive staining was mainly confined in the cytoplasm of cancer cells, and KAI1-positive staining was mainly confined in the membrane and cytoplasm of cancer cells. These data were compared to clinicopathological characteristics. The positive rate of MACC1 protein expression was 60.3 % (196/325) in GAC tissues and 9.2 % (30/325) in normal gastric tissues (Fig. 1a, b), and this difference was shown to be statistically significant (*P* < 0.01). There were also significant differences between the expression of MACC1 and tumor grade (*P* = 0.009), size of tumor (*P* = 0.009), invasion of depth (*P* < 0.001), lymph node metastasis (LNM) (*P* < 0.001), and tumor-node-metastasis (TNM) (*P* < 0.001). In contrast, there were no associations detected between MACC1 expression and patient age (*P* = 0.295), gender (*P* = 0.482), location (*P* = 0.072), and gross type (*P* = 0.108).

In contrast to MACC1 expression, the expression of KAI1 was significantly lower in GAC than in control tissues, with positive rates of 41.2 % (134/325) and 92.3 % (300/325), respectively (*P* < 0.01) (Fig. 1c, d). There were also negative associations between expression of KAI1 in GAC and tumor grades (*P* = 0.045), invasion of depth (*P* < 0.001), lymph node metastasis (*P* = 0.016), and TNM stage (*P* < 0.001). There were no relationships detected between KAI1 expression and patient age (*P* = 0.079), gender (*P* = 0.065), size of tumor (*P* = 0.354), location (*P* = 0.372), and gross type (*P* = 0.965) (Table 2). Spearman correlation coefficient analysis demonstrated a negative correlation between the expression of MACC1 and KAI1 (r = −0.240, *P* < 0.001) (Table 2).

**Table 2:** The association between the expression of MACC1 or KAI1 and clinicopathological characteristics in gastric adenocarcinoma

Variable	MACC1		*P* value	KAI1		*P* value
	Negative	Positive		Negative	Positive	
Gender			0.482			0.065
Male	82	132		118	96	
Female	47	64		73	38	
Ages			0.295			0.079
<58	51	89		90	50	
≥58	78	107		101	84	
Gross type			0.108			0.965
Polypoid	20	17		21	16	
Ulcerative	80	140		130	90	
Invasive	29	39		40	28	
Location			0.072			0.372
Antrum	64	99		92	71	
Cardia	41	77		69	49	
Pylorus	24	20		30	14	
Size			0.009			0.354
*D* < 4.0 cm	35	40		42	33	
4.0 cm ≤ *D* < 8.0 cm	87	124		122	89	
8.0 cm ≤ *D*	7	32		27	12	
Depth of invasion			<0.001			<0.001
Submucosa	13	8		9	12	
Subserosa	53	47		43	57	
Visceral peritoneum	59	125		122	62	
Adjacent structures	4	16		17	3	
Tumor grade			0.009			0.045
Well	20	27		21	26	
Moderate	91	113		120	84	
Poor	18	56		50	24	
Lymph node metastasis			<0.001			0.016
No	93	85		94	84	
Yes	36	111		97	50	
TNM stage			<0.001			<0.001
I and II	96	57		71	82	
III and IV	33	139		120	52	
KAI1^a^			<0.001			
Negative	57	134				
Positive	72	62				

^a^Negative relationship (*r* = −0.240, *P* < 0.001)

### Univariate analysis

**Fig. 2:**
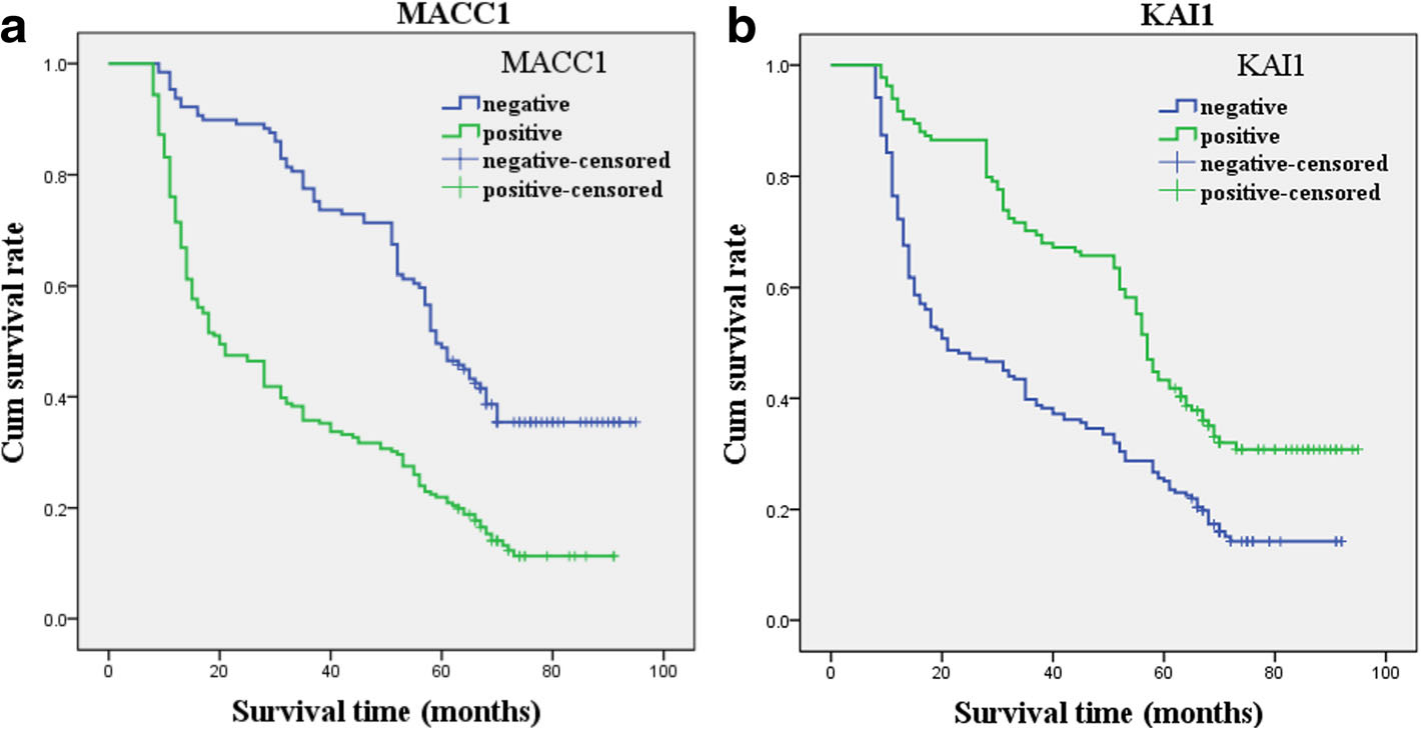
Kaplan-Meier survival analysis by MACC1 and KAI1 status. The *y*-axis represents the percentage of patient; the *x*-axis, their survival in months. The *green line* represents patients with positive expression of MACC1 (**a**) or KAI1 (**b**) with a trend of worse or better survival time than the *blue line* representing the negative MACC1 group or KAI1 group (*P* < 0.001). Mean survival time was 32.7 months for the positive expression of the MACC1 group and 56.1 months for the negative MACC1 group. Mean survival time was 52.6 months for the positive expression of the KAI1 group and 34.5 months for the negative KAI1 group (*n* = 325)

Follow-up data indicated that overall survival time was significantly decreased in GAC patients with positive expression of MACC1 (32.7 months) compared to those who were MACC1-negative (56.1 months) (log-rank = 46.375, *P* < 0.001) (Fig. 2a). On the contrast, the OS time of KAI1-positive patients (52.6 months) was significantly longer than those tumors which were negative (34.5 months) (log-rank = 25.422, *P* < 0.001) (Fig. 2b). In the univariate analysis, OS time was significantly related to clinicopathological characteristics, such as tumor diameter (*P* = 0.033, log-rank = 6.844), invasion of depth (*P* < 0.001, log-rank = 26.806), LNM (*P* < 0.001, log-rank = 75.925), and TNM stage (*P* < 0.001, log-rank = 158.587) (Table 3).

**Table 3:** Results of univariate analyses of overall survival (OS) time

Variable	*n*	Mean OS (months)	Log-rank	*P* value
MACC1			46.375	<0.001
Negative	129	56.1 ± 22.3		
Positive	196	32.7 ± 24.5		
KAI1			25.422	<0.001
Negative	191	34.5 ± 25.6		
Positive	134	52.6 ± 23.4		
Gender			0.187	0.666
Male	214	42.5 ± 26.2		
Female	111	41.0 ± 26.5		
Ages			0.420	0.517
< 58	140	41.6 ± 27.2		
≥ 58	185	42.3 ± 25.6		
Gross type			0.541	0.763
Polypoid	37	46.3 ± 26.8		
Ulcerative	220	41.7 ± 26.1		
Invasive	68	40.5 ± 26.5		
Location			5.536	0.063
Antrum	163	41.4 ± 26.5		
Cardia	118	44.3 ± 26.7		
Pylorus	44	37.9 ± 23.8		
Size			6.844	0.033
*D* < 4.0 cm	75	49.8 ± 27.8		
4.0 cm ≤ *D* < 8.0 cm	211	40.0 ± 25.5		
8.0 cm ≤ *D*	39	38.4 ± 25.0		
Depth of invasion			26.806	<0.001
Submucosa	21	64.1 ± 16.7		
Subserosa	100	50.6 ± 27.1		
Visceral peritoneum	184	35.8 ± 24.3		
Adjacent structures	20	32.5 ± 23.6		
Tumor grade			2.576	0.276
Well	47	47.0 ± 32.6		
Moderate	204	40.8 ± 24.3		
Poor	74	42.0 ± 26.9		
LNM			75.925	<0.001
No	178	53.4 ± 24.1		
Yes	147	28.2 ± 21.8		
TNM stage			158.587	<0.001
I and II	153	60.9 ± 18.1		
III and IV	172	25.2 ± 20.2		

### Multivariate analysis

Multivariate analysis demonstrated that positive expression of either MACC1 or KAI1, as well as TNM stage, was an independent prognostic indicator for GAC (Table 4).

**Table 4:** Results of multivariate analyses of overall survival (OS) time

Covariate	B	SE	*P*	HR	95 % CI
TNM stage	1.277	0.181	<0.001	3.585	2.513–5.112
MACC1	0.385	0.150	0.010	1.470	1.096–1.972
KAI1	−0.434	0.141	0.002	0.648	0.492–0.854

## Discussion

Gastric adenocarcinoma (GAC) is a highly heterogeneous tumor. This heterogeneity may affect the reproducibility of biomarker evaluation [5, 30]. So, thorough investigation of the metastatic and prognostic values of a candidate biomarker is thus required to ensure validity. In our study, we investigated MACC1 expression in GAC and matched normal tissues from 325 patients and compared it to clinicopathological characteristics. We found that MACC1 expression was significantly higher in GAC tissues than that in the control tissues. Furthermore, MACC1 expression was positively correlated with tumor size, grade, invasion of depth, LNM, and TNM stage. Our results were consistent with those of previous studies in GAC [8, 31–33] demonstrating that MACC1 should be useful as a clinical candidate biomarker of GAC.

KAI1, a cell membrane protein that binds to ECM or adhesion proteins [34, 35], is widely considered as a suppresser gene of metastasis in many cancers [19–26]. KAI1 has been correlated with carcinogenesis [20] and showed to predict a poor metastasis and prognosis [19–26]. In this study, we also found that KAI1 expression was significantly related to tumor grade, invasion of depth, LNM, and TNM stage. In addition, Kaplan-Meier survival analysis indicated that GAC patients with positive KAI1 expression had significantly increased survival time compared to those with negative KAI1. These results indicated that KAI1 should play a key role in tumorigenesis, invasion, metastasis, and prognosis of GAC. Several other immunohistochemical studies that investigated the metastatic and prognostic significance of KAI1 in GAC patients obtained similar results [24, 36, 37]. Thus, our results supported the conception that KAI1 should be a credible biomarker of GAC, especially for predicting metastasis and prognosis of cancers.

Metastasis and recurrence are the most common reasons of cancer-related deaths in GAC. TNM staging system is well-known as the guide for devising therapeutic strategies for patients with GAC. However, the TNM staging system cannot provide comprehensive information about the biological behavior of the cancer. Thus, it is urgent to seek novel and effective metastatic and prognostic biomarkers to predict biological behavior (metastasis and recurrence) in GAC patients. In our study, multivariate Cox model analysis showed that the positive expression of either MACC1 or KAI1, as well as TNM stage, was an independent prognostic indicator for patients with GAC.

In our study, we found that MACC1 expression was negatively correlated with KAI1 expression. Furthermore, we also found that there was a negative correlation between the high expression of MACC1 and low expression of KAI1 in the same GAC patient. Abnormal (decreased or lost) expression of KAI1 may be involved in the initiation, development, invasion, metastasis, and recurrence of GAC through lost of function of tumor suppressor gene or suppressor gene of tumor metastasis. Indeed, KAI1, as a suppressor of tumor metastasis, could inhibit β-catenin tyrosine phosphorylation and stabilize E-cadherin-β-catenin complexes to suppress tumor metastasis [38]. In addition, KAI1 could inhibit the process of β-catenin-mediated EMT to prevent tumor angiogenesis and lymphangiogenesis [39]. Meanwhile, MACC1 could be bound to the promoter of the MET gene and activate the HGF/MET signaling pathway to promote cancer cell proliferation, invasion, and metastasis [3, 4]. Furthermore, it could promote angiogenesis and lymphangiogenesis to lead to cancer cell invasion and metastasis [40, 41]. Moreover, KAI1 is able to be bind to c-MET to form a complex or quench the activation of HGF, thus preventing the activation of MACC1 to inhibit the migration of tumor cells [42, 43]. Decreased or lost expression of KAI1 might lose inhibiting the activation of MACC1, angiogenesis and lymphangiogenesis, and stabilization of E-cadherin-β-catenin complexes to promote cancer cell invasion and metastasis. At the same time, abnormal expression of MACC1 could further promote cancer cell invasion and metastasis. However, the methodology of subjects in our study was relatively simple; further studies with more methodologies (such as assessing the effect of target molecules on biological properties in vitro and in vivo models) are needed to verify the present observation.

## Conclusions

Our findings indicate that abnormal expression of MACC1 and KAI1 should play key roles in the development of GAC. The combined detection of MACC1 and KAI1 may be valuable as biomarkers for metastasis and thereby prognosis for patients with GAC.

## Abbreviation

AJCC: American Joint Committee on Cancer
ECM: Extracellular matrix
EMT: Epithelial-mesenchymal transition
GAC: Gastric adenocarcinoma
HGF: Hepatocyte growth factor
KAI1: Kangai 1
LNM: Lymph node metastasis
MACC1: Metastasis-associated in colon cancer-1
MET: Mesenchymal-epithelial transition
OS: Overall survival
TM4SF: Transmembrane 4 superfamily
TNM: Tumor node metastasis
WHO: World Health Organization
